# New Materials and Structures: Anti-Escape Trap Net for Trapping *Eucryptorrhynchus brandti* (Harold, 1880) (Coleoptera: Curculionidae)

**DOI:** 10.3390/insects15110857

**Published:** 2024-11-02

**Authors:** Hongyu Li, Weicheng Ding, Chao Wen, Junbao Wen

**Affiliations:** 1 State Key Laboratory of Efficient Production of Forest Resources, Beijing Forestry University, Beijing 100083, China; hongyuli@bjfu.edu.cn (H.L.); weichengding@bjfu.edu.cn (W.D.); 2 Beijing Key Laboratory for Forest Pest Control, Beijing Forestry University, Beijing 100083, China; 3 Research Center for Forestry Pest Risk Analysis, Beijing Forestry University, Beijing 100083, China; 4 College of Grassland Science, Beijing Forestry University, Beijing 100083, China; wenchao@bjfu.edu.cn

**Keywords:** *Eucryptorrhynchus brandti*, anti-escape trap net, pest management, physical control

## Abstract

The weevil *Eucryptorrhynchus brandti* (Harold, 1880), a wood-boring pest of *Ailanthus altissima* (Mill.) Swingle, has caused significant ecological damage and economic loss in China. Physical control was effective against the related species *Eucryptorrhynchus scrobiculatus* (Motschulsky). One type of trunk trap net controlled the adult weevil population through blocking and trapping. However, the effectiveness of this device was compromised by their tendency to escape when encountering such trapping nets. Therefore, it is crucial to enhance and optimize both the material composition and structural design of trap nets to enhance weevil capture rates by mitigating escape behavior. In this study, we compared the capture efficacy of an anti-escape trap net (AETN) with novel materials (Velcro) and a double-layer trap net (DLTN). The results indicate that the mean recapture rate of AETNs (50%) was significantly higher than that of DLTNs (3%) in laboratory tests. The total number of *E. brandti* captured using AETNs was 11 times higher than that of DLTNs in field marker recapture tests and 4 times higher in capture tests on field populations. The new AETN structures could effectively prevent the escape behavior of *E. brandti* and capture weevils. The use of Velcro made the AETN easier to manufacture, more convenient to use, and less costly. Overall, the AETN is an economical and environmentally friendly physical control device.

## Simple Summary

This research primarily targets a severely harmful species of weevil, *Eucryptorrhynchus brandti*. By investigating its escape and ascending–descending behaviors, this study reveals that physical control represents an effective prevention and control method. Building upon previous studies, researchers developed a novel anti-escape trap net. The anti-escape trap net was very efficient in the prevention and control of this species and also features innovations in structure and materials that enable its wide application in the management of other pests. The anti-escape trap net constitutes a cost-efficient and environmentally sustainable approach to physical pest prevention and control.

## 1. Introduction

**Figure 1:**
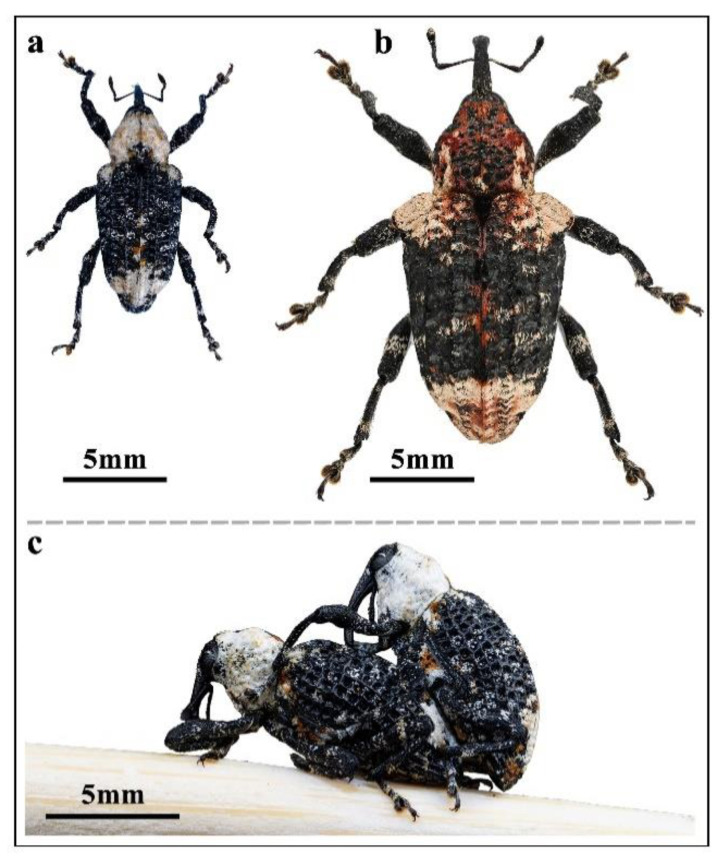
Images of adult morphology: (**a**) *E. brandti*; (**b**) *E. scrobiculatus*; (**c**) male and female of *E. brandti* holding pair.

*Eucryptorrhynchus brandti* (Harold, 1880) (Coleoptera: Curculionidae) is a wood-boring pest that exclusively harms the tree of heaven, *Ailanthus altissima* (Mill.) Swingle. The weevil is related to *Eucryptorrhynchus scrobiculatus* (Motschulsky, 1854) (Figure 1) [1,2]. As a native plant in China, the tree of heaven is characterized by strong adaptability. It can grow in areas with drought and blown sand and has high ornamental value [3]. It has large cultivation areas in most areas of China and is an important afforestation pioneer [4]. The tree is considered to be an invasive species in North America and certain parts of Europe [5,6]. The presence of allelopathic chemicals that can inhibit the growth of local plants contributes to strong competitiveness [7]. The introduction of two species of weevils is considered the most promising biological control measure to control the propagation of tree of heaven [8].

The two pests harm different parts of the host and have obvious niche differentiation. The larvae of *E. scrobiculatus* primarily feed on the tree roots and emerge from the soil, while adults ascend the tree to supplement nutrition by feeding on young branches or petioles, resulting in tree debilitation [9]. The larvae of *E. brandti* primarily feed on the phloem and emerge from the trunk, thereby causing extensive damage to the conducting tissue and ultimately resulting in death [10]. Adult *E. brandti* overwinters at the base of the trunk and in the crevices of the soil. The following year, overwintering adults return, ascending to the tree to replenish nutrients [11]. The adults of the two species display a specific habit of ascending and descending on the trunk [12].

Environmental problems may result from chemical pest-control measures used to control *E. scrobiculatus* and *E. brandti*. Physical control is more environmentally friendly and further promotes research on physical control devices [13,14]. For the physical control of *E. scrobiculatus* and *E. brandti*, Yang et al. designed a trunk trap net (TTN) that significantly reduced the population of *E. scrobiculatus* [12]; an adhesive trunk trap net (ATTN) capturing a small number of *E. brandti* [15]; and overwintering traps (PETBT and CPBT) for overwintering weevils [16]. However, existing physical control devices are not effective in capturing *E. brandti*, and there are many problems with the application of physical control devices in field environments. Therefore, the population of *E. brandti* is not controlled, and the ecological benefits of tree-of-heaven planting areas are seriously affected. Further research is needed to improve the capture efficiency of trap nets so as to reduce the damage caused by weevils to tree of heaven.

Through extensive field observations and experiments, various studies have been conducted on the behavior of *E. brandti*, including oviposition behavior [17], feeding behavior [11], death-feigning behavior [18], escape behavior [12], and aggregation behavior [19]. Studying insect behavior is of great value in developing physical control devices. Yang et al. compared the escape behaviors of *E. brandti* and *E. scrobiculatus* in a TTN in a laboratory experiment [12,15]. Through screening the mesh of the TTN and the application of entomological glue (CIXI QIXINGQIAO ADHESIVE Co., Ltd., CiXi, China), weevils were obstructed and captured. However, there were problems with its practical application in the field. Therefore, to counter the escape behavior of *E. brandti* and to be efficient and convenient in practical production and application, the physical control device for *E. brandti* needs to be improved and innovative.

By building upon previous research on TTNs, this study further explored the behavior of weevils ascending and descending trees and their interaction with TTNs through comprehensive field observations and laboratory experiments to address the existing challenges associated with the use of TTNs. In this study, we developed two new trap nets based on a previous TTN with improved materials and structures. The first is a double-layer trap net (DLTN), which increases the density of the net to improve the capture effect. The second is an anti-escape trap net (AETN), which prevents the escape behavior of weevils and completely obstructs the escape route. We evaluated the capabilities of both trap nets in laboratory and field trials. The use of new materials and structures will enable this physical capture device to achieve both environmentally friendly and efficient pest-control objectives.

## 2. Materials and Methods

### 2.1. Collection and Feeding of E. brandti

Adult weevils were collected from the trunks, branches, and bases of trees in a field (Lingwu City, Ningxia Autonomous Region, China). The collected adults were kept in rearing boxes with fresh tree branches that were replaced daily. The tests were conducted after 3 days of rearing.

### 2.2. Experimental Sites

**Figure 2:**
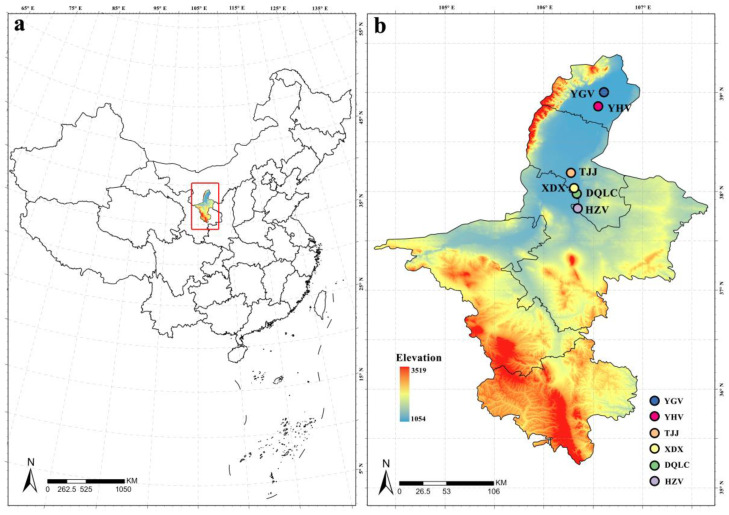
(**a**) Ningxia Hui Autonomous Region in China; (**b**) six experimental plots are marked with different colors (elevation is in meters).

Sixexperimental sites were selected from within the following plantations of *A. altissima*: Daquan Forestry Farm (DQFF) (37°58′35″ N, 106°19′59″ E); Haizi Village (HZV) (37°49′42″ N, 106°20′33″ E); TaoJiaJuan (TJJ) (38°11′17″ N, 106°16′27″ E); Xinduxian (XDX) (38°2′0″ N, 106°18′24″ E), located in Lingwu; Yanhe Village (YHV) (38°51′40″N, 106°33′0″ E); and Yinguang Village (YGV) (39°0′15″ N, 106°36′30″ E), located in ShiZuiShan. All of them are located in the north-central Ningxia Hui Autonomous Region in China (Figure 2). The three sites TJJ, YHV, and YGV are farmland shelterbelts, and the other three sites are on both sides of the canal. The sites have temperate continental arid and semi-arid climate, with an average annual temperature of 8.4–9.9 °C and annual rainfall of 150–300 mm. The temperature difference between day and night during summer is 12–15 °C.

### 2.3. Observe Adults’ Ascending–Descending Behavior on the Tree

**Figure 3:**
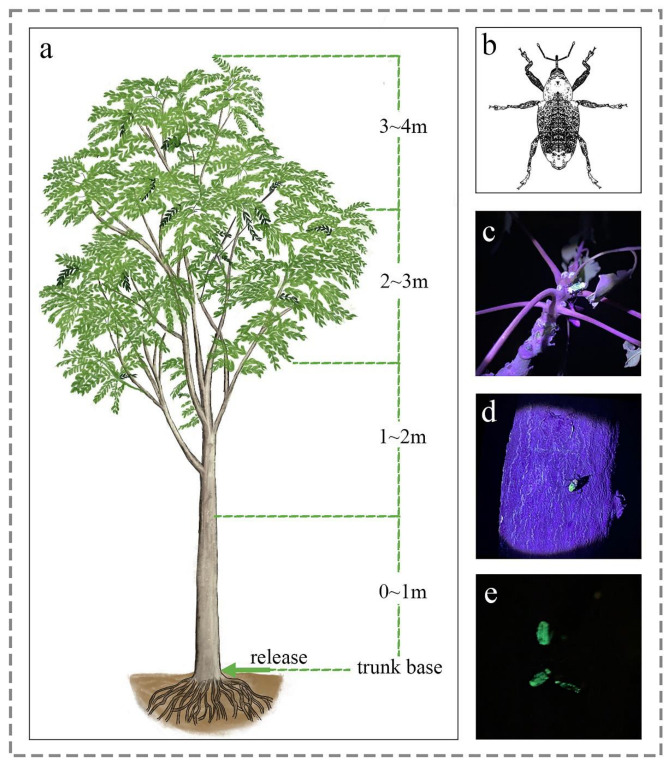
Observation of the ascending–descending behavior of *E. brandti* on the tree: (**a**) the diagram of different parts of the tree that were observed, with the release site located at the base of the trunk; (**b**) a schematic diagram of *E. brandti*; (**c**) the weevil stayed at the treetops, under the ultra-violet flashlight at night, the weevil painted with fluorescent paint was green; (**d**) the weevil stayed at the tree trunk; (**e**) the weevil stayed at the base of the trunk.

Observing the ascending–descending behavior of weevil trees through experiments can provide useful information for developing trap nets. A healthy *A. altissima* tree (height = 4.23 m, diameter = 13.2 cm) was selected from the experimental site and transplanted into an open experimental site with no other trees. The experimental tree was divided into five observation parts: the trunk base (including the surrounding soil), 0–1 m trunk, 1–2 m trunk, 2–3 m branches, and 3–4 m treetop (Figure 3a). Fifty adult weevils (*E. brandti*) were randomly selected and marked with yellow, fluorescent paint, and the marked weevils were released at the base of the trunk at 7:00. The number of marked weevils in different parts was counted every hour after release, and the location of the weevils was observed in the dark using an ultra-violet flashlight (365 nm) (Figure 3c–e). The experiment was conducted from 7:00 to 24:00, and three replicate trials were conducted.

### 2.4. Design and Construction of Two Trap Nets

Based on the ascending–descending behavior on the trunk of *E. brandti*, two types of trap nets were designed to capture adults on the tree to control population density and the damage caused by weevils to *A. altissima*.

The first trap net type was a double-layer trap net (DLTN), which consisted of a black nylon elastic rope (length = 120 cm, diameter = 2 mm, GuangZhouZhengXin Accessories Co., Ltd., Guangzhou, China) and a rectangular nylon net (length = 150 cm, width = 60 cm, mesh = 5 mm; Yingchun Fishing Net Factory, Hefei, Anhui Province, China). An elastic rope was passed through the middle of a nylon net, wrapped around the trunk, and tied tightly (Figure S1a).

The second trap net type was an anti-escape trap net (AETN), which consisted of a black Velcro strap (length = 120 cm, width = 1 cm, Quanzhou Ming Qian Weaving Co., Ltd., Fujian, China) and a specially folded rectangular nylon net (length = 150 cm, width = 100 cm, mesh = 5 mm). The AETN was applied by wrapping Velcro around the tree trunk in a circle. It was secured by Velcro pasting front and back. The trap net surrounded the entire trunk (Figure S1b).

### 2.5. Observation of the Interaction Behavior Between Weevils and Trap Net

By observing and judging the interaction behavior between weevils and trap nets, we could compare the effects of the two types of trap nets. Therefore, we conducted a study to document the different behaviors exhibited when *E. brandti* was exposed to the trap nets. Three sections of tree trunks (length = 100 cm, diameter = 20 cm) were felled from the field forest for laboratory testing, and two types of trap nets were prepared. Three sections of tree trunks were placed vertically on the test bench, and an AETN was installed in the top third of the wood section. Adult weevils collected in the field were divided into three groups, i.e., 50 females, 50 males, and 20 groups of male and female holding pairs, meaning the males and females were in a mating position (Figure 1c).

The three groups were placed at the bottom one-third of the three tree trunks, and the weevils were guided to crawl by blowing air from the bottom to the top with a rubber suction bulb. The observation time was one hour to determine whether there was an interaction between the weevils and the trap net. The experimental procedures for weevils and DLTN were the same. The number of different interactions between the weevils and trap nets was counted for further comparative analyses.

### 2.6. Laboratory Test Comparing the Efficacy of Different Trap Nets

Results for the two trap nets were compared in the laboratory based on the ascending–descending behavior of the weevils on the trunk. In the laboratory, we built a frame structure of six cuboids (length = 50 cm, width = 50 cm, height = 200 cm) with wooden strips. The entire wooden frame was covered with a breathable mesh screen with a smaller mesh (mesh = 0.5 mm) to prevent weevils from escaping (Figure S2). Six 1 m long tree trunk segments were collected from the field and placed vertically in the center of a wooden frame, and the top and bottom sections of the trunk were sealed with tape. Trap nets were placed on the trunk one-third of the way from the top to provide the test adults with a sufficient range of movement.

One experimental device was set up in each group, and the different types of trap nets were repeated three times for a total of six groups. After the experimental device was set up, the collected weevils were released at the base of the trunk segment, and 100 weevils were released from each group. The number of weevils caught in each net was counted after 24 h.

### 2.7. Field Mark–Release–Recapture Trials with Different Trap Nets

Mark–release–recapture trials were used to eliminate experimental errors caused by fluctuations in the population size of the weevil in the field and differences in population size and density among sites. Field trials were conducted at three locations: DQFF, HZV, and TJJ. The test trees with trap nets were selected based on a completely random block design in every field site, and the trap nets were installed on the tree trunks at approximately 1.2 m from the ground. The two treatments (AETN and DLTN) were replicated ten times in each site. Adults reared in the laboratory were marked with yellow, fluorescent paint (Shanxi Fuduo Electronic Technology Co., Ltd., Taiyuani, China) and brought to the test site for manual release at 7:00. For each tree, 30 mixed-sex group weevils were released at the base of the trunk, i.e., 300 weevils were released per block, and 600 weevils were released per site. All treatments were surveyed after a week, and the number of marked weevils captured in each trap net was recorded. At the same site, each treatment was spaced at 10 m intervals within blocks, and the distance between neighboring blocks was greater than 50 m. The experiment was conducted from July to September 2020 and was repeated once a month, for a total of three times.

### 2.8. Different Types of Trap Net Capture Wild Weevils

Experiments were conducted to compare the capture effects of two trap nets on wild weevil populations. Two treatments (AETN and DLTN) were applied, as described in Section 2.7. Randomized complete block design trials were performed at each site, with ten replicate blocks. Each trap net was randomly assigned to 10 trees in each block, with 20 trees at each site. The distance between neighboring blocks was greater than 50 m. All treatments were surveyed after two weeks, and the number of captured weevils in each trap net was recorded. Field trials were conducted at three locations, i.e., XDX, YHV, and YGV, from July to September 2021.

### 2.9. Data Analysis

The Shapiro–Wilk test was used to test for the normal distribution of all dependent variables according to the sample size, and the Levene test was performed for homoscedasticity [20]. These data could not be transformed to satisfy the assumptions of the parametric tests, including weevil recapture rates, the number of weevils in different parts of the trunk, and the interaction behavior between weevils and trap nets, and were compared using the non-parametric Kruskal–Wallis test. In the laboratory experiments, weevil catch data were log (x+1)-transformed to achieve normality and homoscedasticity, and different treatments were compared using Tukey’s test. The mixed-model analysis of variance (ANOVA) was used to analyze the field test data, with blocks within sites considered a random factor and different treatments, sites, and their interactions considered fixed factors. All data were analyzed by applying IBM SPSS Statistics 26 at the α = 0.05 level.

## 3. Results and Analysis

### 3.1. Ascending–Descending Behavior on the Trunk

**Figure 4:**
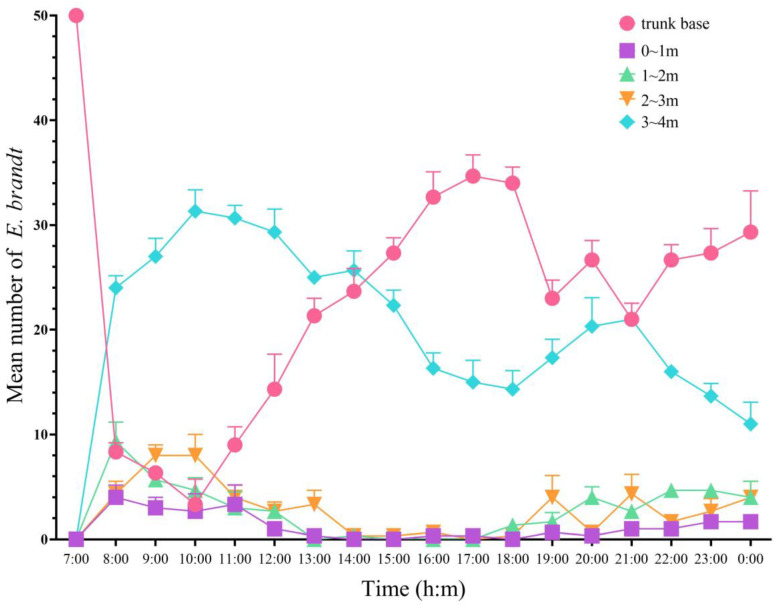
The distribution of adults in different parts of the tree in different time periods.

Weevils ascended and descended on the trunk at different times in certain patterns. The number of adults in different parts of the tree varied significantly (F = 43.862, df = 4, *p* < 0.001). The number of adults on the trunk base and treetop changed greatly during different time periods and formed an obvious contrast. The number of adults at the base of the trunk was the lowest at 10:00 and highest at 17:00; the number of adults at the treetop was the highest at 10:00 and lowest at 0:00 (Figure 4).

### 3.2. The Interaction Behavior between Weevils and Trap Nets

Based on observations during the experiments, five types of behavior of weevils interacting with trap nets were identified: (1) **No Contact:** weevils did not contact the trap net, always in other areas of the trunk outside the trap net; (2) **Exit after Entry:** weevils entered the trap net but subsequently exited following their original route after a period of time; (3) **Climbing and Escape:** the weevils climbed onto the outermost surface of the net through the lower edge and escaped to the upper part of the tree trunk; (4) **Escape from Net Mesh**: after entering the net, they continuously struggled through the net to escape; and (5) **Capture by the Net:** the legs and bodies of the weevils became entangled in the net, resulting in their capture.

Upon comparing the interaction behaviors of the two types of trap nets with weevils, the results indicated that the total number of adults escaping from the DLTN was 7.11 times greater than that observed in the AETN. The total number of escapes comprised those climbing and escaping as well as those resulting from escape from net mesh. Additionally, the total number of weevils captured by the AETN was 3.12 times higher than those captured by the DLTN.

**Figure 5:**
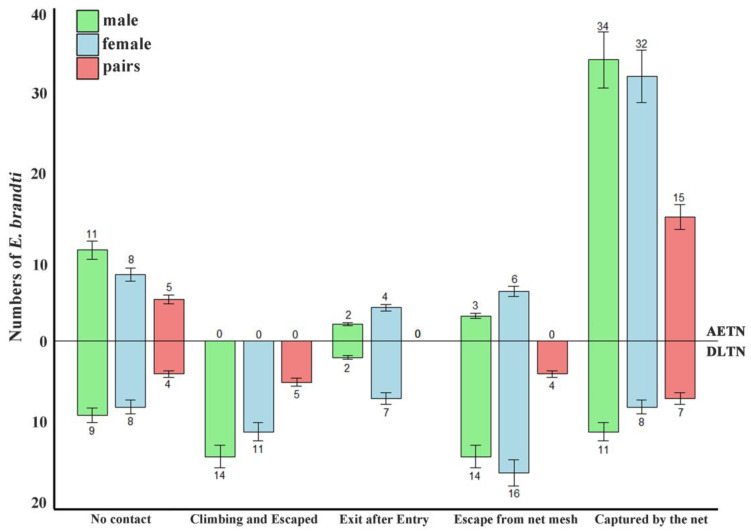
Observation of interaction behavior between weevil and trap nets (mean ± S.E.).

Significant differences were observed in the number of different behaviors among the three groups—females, males, and holding pairs—when DTN was applied (F = 9.00, df = 2, *p* = 0.009), whereas no significant difference was found in the number of different behaviors among the three groups when AETN was applied (F = 3.38, df = 2, *p* = 0.086) (Figure 5).

### 3.3. Capture Effect of Two Trap Nets in the Laboratory

**Figure 6:**
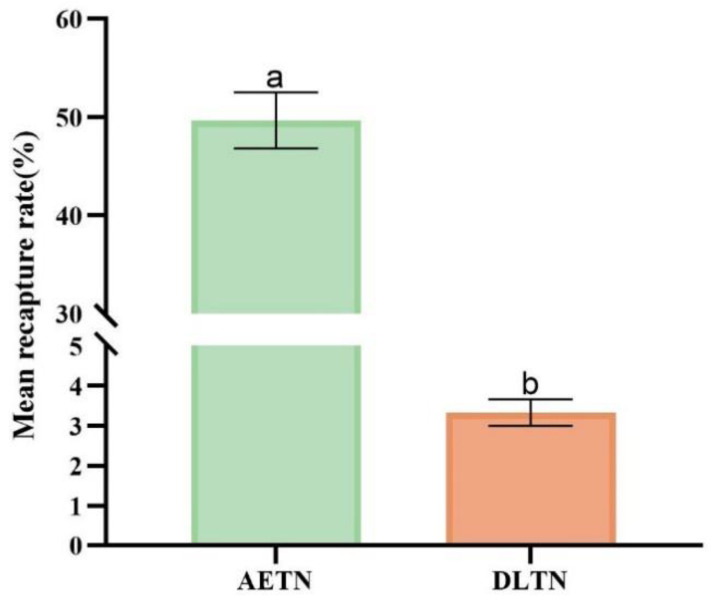
Comparison of the recapture rate (mean percentage ± S.E.) of two types of trap nets in the laboratory. Columns with the same letter were not significantly different (Kruskal–Wallis test α = 0.05).

A comparative analysis was performed of the recapture efficacy between two types of trap nets, AETN and DLTN, which were deployed to capture weevils within a laboratory setting. The findings revealed that the mean recapture rate of AETN was 49.67%, whereas that of DLTN was 3.33%. Additionally, statistical analysis indicated a significantly higher mean recapture rate for AETN than for DLTN (F = 3.971; df = 1; *p* = 0.046; Figure 6).

### 3.4. Capture Effect of Two Trap-Net Types with Field Marker Release

**Figure 7:**
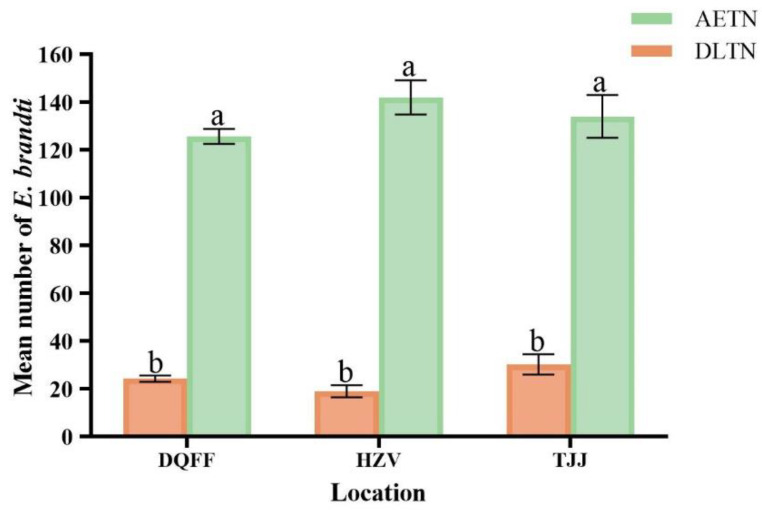
The number (mean ± S.E.) of adult weevils released from markers captured by the two trap nets. Columns with the same letter were not significantly different (Tukey’s HSD, ± = 0.05).

The total number of weevils captured across the three sampling sites (HZV, TJC, and DQF) was 1100 for AETN and 96 for DLTN. The results indicated that a significantly higher number of weevils were caught using AETN than by DLTN across the different sample sites. The marker recapture rates of AETN in HZV, TJC, and DQF were 63.2, 62, and 58.2%, respectively, whereas those of DLTN in the same sample plots were 6.3, 5.5, and 4.2%, respectively (Figure 7). The treatments significantly affected the number of captures, and the interaction between the sample site, treatment, and sample site had no significant effect on the number of captures (Table 1).

### 3.5. Capture Effect of Two Trap Nets for Field Populations

**Figure 8:**
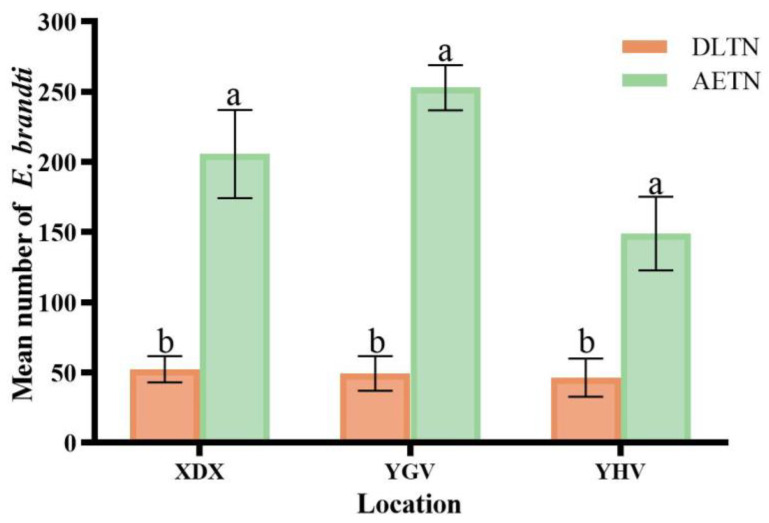
Two kinds of trap nets capture populations of weevil in the wild (mean ± S.E.). Columns with the same letter were not significantly different (Tukey’s HSD, ± = 0.05).

The total number of weevils captured across the three sampling sites (XDL, YGV, and YHV) was 1823 for AETN and 444 for DLTN. A significantly higher number of weevils were caught using AETN than by DLTN at different sampling sites (XDL: t = 4.676, df = 4, *p* = 0.09; YGV: t = 10.078, df = 4, *p* = 0.01; YHV: t = 3.468, df = 4, *p* = 0.03). Specifically, the number of weevils caught using AETN in XDL was 4 times higher than that caught using DLTN, while it was 5 times higher in YGV, and 3.2 times higher in YHV (Figure 8).

Treatments significantly influenced the number of captures. The interaction between treatment and sample sites had no significant effect on the number of captures (Table 1).

## 4. Discussion

Our study described the process that the *E. brandti* has an obvious ascending–descending behavior on the tree and displays two different escape behaviors when interacting with the trap net. Based on behavioral studies, an AETN was developed for *E. brandti*. We found that (1) the structure of AETN made it easier to capture the weevil and effectively prevented it from escaping; (2) AETN uses Velcro as an attachment material, which prevented the weevil from escaping and is more convenient and fast in application; (3) the capture effect of AETN was significantly higher than that of DLTN; and (4) it significantly reduced the labor cost of AETN production and application.

The regularity observed in insect behavior is of the utmost importance in investigating pest-control methods. The ascending–descending behavior of weevils on trees has served as a crucial foundation for developing physical control devices. In this study, we observed that *E. brandti* exhibited heightened activity at both the canopy and the base of the trunk, thereby corroborating the behavioral patterns associated with the feeding habits of *E. brandti* and avoidance behavior toward the tree.

This study revealed escape behavior when TTN was used to control both weevil species. TTN is effective in controlling *E. scrobiculatus* but does not effectively manage *E. brandti* [12,15]. The main reason for this was that *E. brandti* is smaller and can easily escape from the surface and mesh of the trapping net. Insects instinctively exhibit escape behavior when threatened by predators or other disturbances. Locusts can visually orient themselves and quickly switch between walking, flying, and jumping when escaping predators. The escape behavior of locusts differs among different species [21]. The study of escape behavior in ants is particularly intriguing as ants exhibit a systematic response when disturbed by external stimuli, thereby providing valuable insights for comparing human evacuation strategies during emergency situations [22,23]. Therefore, the main research focus has been on developing effective strategies to prevent pests from escaping or avoiding the use of physical control devices. In the laboratory experiment, three treatments were conducted based on the three groups—female, male, and holding pair—and their interactions with the trapping nets were examined. These results indicated that AETN successfully countered the escape behavior of *E. brandti*. Among these treatments, the trapping effect on mating pairs was significant. This was primarily due to the larger combined size and slower movement behavior of the paired individuals. This treatment was chosen based on previous research findings indicating that *E. brandti* could engage in multiple mating sessions per day, each lasting more than 10 min [24]. This information also serves as a useful reference for the timing of trapping net placement before the breeding peak.

Physical control devices are often used in combination with insecticides. The insecticide bifenthrin-treated netting (LLITN) has been employed by researchers to manage whiteflies and thrips while also assessing its impact on the natural enemies of these pests. The findings of this study demonstrate that LLITN exhibits favorable compatibility with natural enemies, thus establishing it as a valuable tool for integrated pest management (IPM) [25]. However, employing purely physical methods such as trap nets for enhanced control is not commonly practiced. Studies have shown that the use of exclusion netting in vineyards to control *Lycorma delicatula* (White, 1845) (Hemiptera: Fulgoridae) is effective, and the use of huge barrier nets to cover the entire row of vines reduces the use of pesticides despite the high cost [26]. Recently, significant advances have been made in the physical control of *E. brandti* and *E. scrobiculatus*. These include the implementation of adhesive tape on tree trunks to effectively impede weevil climbing; ground-trap devices designed to capture emerging *E. scrobiculatus* from the soil [27]; and trunk trap nets (TTNs) [12], adhesive trunk trap nets (ATTNs) [15], and overwintering traps [16]. There are many problems with the actual field applications of ATTN. Through investigation, it was found that the sticky insect glue could not be evenly applied to the trap nets, some of which were attached to tree trunks and could not block the weevils. Moreover, with prolonged exposure to the outdoor environment, the adhesive gradually decreased in stickiness, and the cost of replacing ATTN once per month was too high. In conclusion, there is currently no definitive method for effectively controlling weevils, leading to escalated damage caused by this pest.

In response to the problems encountered in controlling *E. brandti*, a DLTN was designed using a TTN and an ATTN to capture weevils by increasing the density of the net. However, the capture effectiveness of the DLTN was unsatisfactory in both indoor and field tests. This can be attributed to the significant gap formed between the elastic rope and net when applied to the tree trunks. *E. brandti* easily escaped from positions close to the elastic rope, whereas some weevils managed to evade capture by escaping from the surface of the trap net.

**Figure 9:**
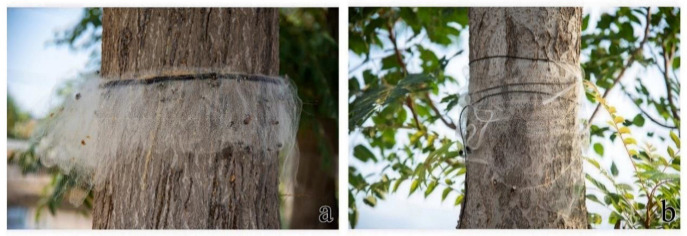
Two types of trap net field practical application images: (**a**) anti-escape trap net (AETN); (**b**) double-layer trap net (DLTN).

**Figure 10:**
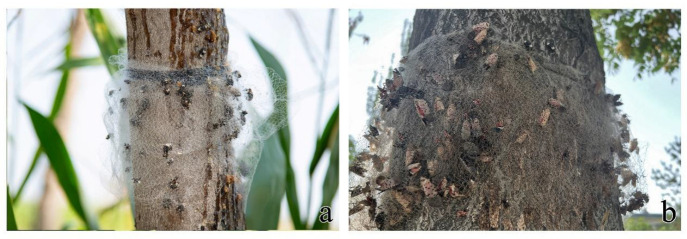
Insects captured by AETN in the plantation: (**a**) *E. brandti* and *E. scrobiculatus*; (**b**) *L. delicatula*.

We developed an AETN and compared it with a DLTN (Figure 9a,b) to address these challenges. The AETN was developed by replacing an elastic rope with a novel material called Velcro. Velcro possesses double-sided mutual adhesion and lacks elasticity, which ensures that it does not compromise the elasticity or structure of the net when fixed to the trunk. Moreover, affixing the device directly around the trunk eliminates the need for knotting. The key characteristic of the AETN lies in its formation of a three-dimensional spatial structure through a specifically folded trap net, which effectively establishes an extensive barrier range on the trunk upon application, thereby preventing weevils from escaping through the surface of the trap net. Furthermore, the internal multilayer folding structure hinders weevil escape from the mesh and deprives them of support from the trunk when crawling along the AETN toward the edge of the trap net, making it easier for the weevil to entangle its feet and body in the net. The results of both laboratory and field tests demonstrated that the AETN exhibited a significantly higher capture efficiency than the DLTN. In the field experiments, AETN captured a significant number of *E. scrobiculatus* and *Lycorma delicatula* (Figure 10a,b). Furthermore, AETN can be manufactured in rolls, so that the trap net can be cut to the exact diameter of the trunk. In contrast, a one-size-fits-all DTN and ATTN cannot accommodate all tree diameters, often leading to incomplete coverage and other issues, thus negatively impacting the control of *E. brandti*. The production and application of AETN significantly reduced both labor and time costs compared with other devices.

## 5. Conclusions

Research on control measures for *E. brandti*, an important pest that causes significant damage, has been continuously updated. Physical control remains the most effective approach. The AETN effectively mitigated most instances of *E. brandti* escape. This physical control device is characterized by ease of manufacturing, user convenience, environmental friendliness, and cost-effectiveness. Because of the strong adhesive properties of Velcro, AETN can have a longer lifespan in the field, requiring replacement only once a year if no visible damage is present.

In general, AETN could effectively control the population of *E. brandti* and significantly mitigate its impact on *Ailanthus altissima*, thereby serving as an environmentally friendly physical control measure. Future studies should focus on assessing the efficacy of large-scale AETN applications and exploring their potential against other wood-boring pests.

## Figures and Tables

**Table 1:** Difference analysis of the effects of different trap nets and plots on the catches of weevils.

Sources of Variation	Mark–Release–Recapture Field Trials			Capture Trials of Wild Population		
	df	F	*p*	df	F	*p*
Treatments	1	441.088	<0.001	1	89.527	<0.001
Locations	2	1.54	0.254	2	3.674	0.57
Treatments * Locations	2	0.669	0.53	2	3.242	0.75

* means interaction “Treatments × Locations”.

## Data Availability

Data are contained within the article.

## Supplementary Materials

The following supporting information can be downloaded at: https://www.mdpi.com/article/10.3390/insects15110857/s1, Figure S1: Trap net making steps. (a) double-layer trap net (DLTN); (b) anti-escape trap net (AETN). Figure S2: Laboratory test comparing the efficacy of different trap nets. (a) apply double-layer trap net (DLTN); (b) apply anti-escape trap net (AETN).
