# Supplementary figures and images for: New Materials and Structures: Anti-Escape Trap Net for Trapping Eucryptorrhynchus brandti (Harold, 1880) (Coleoptera: Curculionidae)
Source: Insects. 2024 Nov 2;15(11):857. doi: 10.3390/insects15110857 (PMC11594905; doi:10.3390/insects15110857)

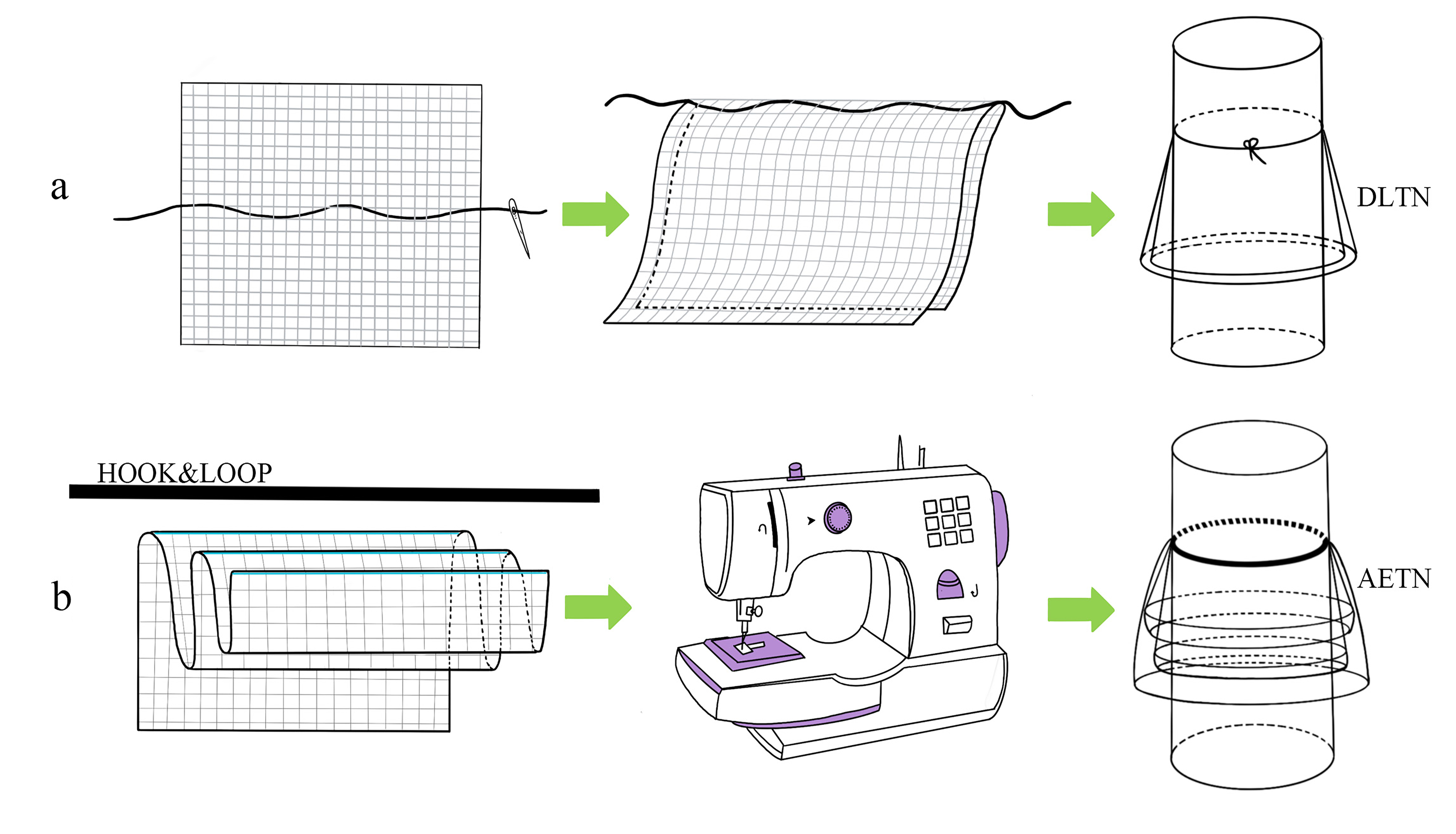

Supplement: Supplementary file 1 [file insects-15-00857-s001.zip › insects-3245625-supplementary/Fig S1.jpg]
